# Predicting long-term neurocognitive outcome after pediatric intensive care unit admission for bronchiolitis—preliminary exploration of the potential of machine learning

**DOI:** 10.1007/s00431-023-05307-3

**Published:** 2023-11-06

**Authors:** Eleonore S. V. de Sonnaville, Jacob Vermeule, Kjeld Oostra, Hennie Knoester, Job B. M. van Woensel, Somaya Ben Allouch, Jaap Oosterlaan, Marsh Kӧnigs

**Affiliations:** 1 Department of Pediatric Intensive Care, Emma Children’s Hospital, Amsterdam UMC location University of Amsterdam, Meibergdreef 9, Amsterdam, The Netherlands; 2 Emma Children’s Hospital Amsterdam UMC Follow Me program & Emma Neuroscience Group, Emma Children’s Hospital, Amsterdam UMC location University of Amsterdam, Meibergdreef 9, Amsterdam, The Netherlands; 3 Amsterdam Reproduction and Development research institute, Amsterdam, The Netherlands; 4 University of Amsterdam, Informatics Institute, Science Park 904, Amsterdam, The Netherlands

**Keywords:** Child, Pediatric intensive care unit, Neurocognition, Machine learning

## Abstract

**Purpose:**

For successful prevention and intervention, it is important to unravel the complex constellation of factors that affect neurocognitive functioning after pediatric intensive care unit (PICU) admission. This study aims (1) to elucidate the potential relevance of patient and PICU-related characteristics for long-term adverse neurocognitive outcome after PICU admission for bronchiolitis, and (2) to perform a preliminary exploration of the potential of machine learning as compared to linear regression to improve neurocognitive outcome prediction in a relatively small sample of children after PICU admission.

**Methods:**

This cross-sectional observational study investigated 65 children aged 6–12 years with previous PICU admission for bronchiolitis (age ≤ 1 year). They were compared to demographically comparable healthy peers (*n* = 76) on neurocognitive functioning. Patient and PICU-related characteristics used for the prediction models were as follows: demographic characteristics, perinatal and disease parameters, laboratory results, and intervention characteristics, including hourly validated mechanical ventilation parameters. Neurocognitive outcome was measured by intelligence and computerized neurocognitive testing. Prediction models were developed for each of the neurocognitive outcomes using Regression Trees, k-Nearest Neighbors, and conventional linear regression analysis.

**Results:**

The patient group had lower intelligence than the control group (*p* < .001, *d* = −0.59) and poorer performance in neurocognitive functions, i.e., speed and attention (*p* = .03, *d* = −0.41) and verbal memory (*p* < .001, *d* = −0.60). Lower intelligence was predicted by lower birth weight and lower socioeconomic status (*R*^2^ = 25.9%). Poorer performance on the speed and attention domain was predicted by younger age at follow-up (*R*^2^ = 53.5%). Poorer verbal memory was predicted by lower birth weight, younger age at follow-up, and greater exposure to acidotic events (*R*^2^ = 50.6%). The machine learning models did not reveal added value in terms of model performance as compared to linear regression.

*Conclusion*: The findings of this study suggest that in children with previous PICU admission for bronchiolitis, (1) lower birth weight, younger age at follow-up, and lower socioeconomic status are associated with poorer neurocognitive outcome; and (2) greater exposure to acidotic events during PICU admission is associated with poorer verbal memory outcome. The findings of this study provide no evidence for the added value of machine learning models as compared to linear regression analysis in the prediction of long-term neurocognitive outcome in a relatively small sample of children.

**Table:** 

**What is Known:** *• Adverse neurocognitive outcomes are described in PICU survivors, which are known to interfere with development in other major domains of functioning, such as mental health, academic achievement, and socioeconomic success, highlighting neurocognition as an important outcome after PICU admission.* *• Machine learning is a rapidly growing field of artificial intelligence that is increasingly applied in health care settings, with great potential to capture the complexity of outcome prediction.*	
**What is New:** *• This study shows that lower birth weight, lower socioeconomic status, and greater exposure to acidotic events during PICU admission for bronchiolitis are associated with poorer long-term neurocognitive outcome after PICU admission. Results provide no evidence for the added value of machine learning models in a relatively small sample of children.* *• As bronchiolitis seldom manifests neurologically, the relation between acidotic events and neurocognitive outcome may reflect either potentially harmful effects of acidosis itself or related processes such as hypercapnia or hypoxic and/or ischemic events during PICU admission. This study further highlights the importance of structured follow-up to monitor long-term outcome of children after PICU admission.*

**Supplementary Information:**

The online version contains supplementary material available at 10.1007/s00431-023-05307-3.

## Introduction

With advances in pediatric intensive care, the survival rate of children admitted to the pediatric intensive care unit (PICU) has increased dramatically in the past decades [1, 2]. Yet, long-term morbidity after PICU admission is a growing concern [3, 4]. Sequelae are described in physical, neurocognitive, and psychosocial health [3–7]. Adverse neurocognitive outcomes are known to interfere with development in other major domains of functioning, such as physical and mental health [8, 9], academic achievement [10], and socioeconomic success (as measured by education, occupation, and income) [11], highlighting neurocognition as an important outcome after PICU admission.

In the literature, multiple pathophysiological mechanisms have been proposed that may contribute to long-term neurocognitive outcome of critically ill patients, including hypoxia, metabolic derangements such as glucose dysregulation and ischemia [12–14]. Such mechanisms may be triggered by the underlying disease [15], the critical deterioration [7, 16], and/or the associated treatments at the PICU [17, 18]. In addition, also demographic characteristics such as age at PICU admission, age at follow-up, sex, and socioeconomic status have been found related to neurocognitive outcome after PICU admission [7, 18–20]. As understanding of the origin of difficulties in neurocognitive functioning is a prerequisite for successful prevention and intervention, it is important to unravel the factors that affect neurocognitive functioning after PICU admission.

Digitalization of health care provides increasingly more data that can importantly contribute to better prediction and understanding of long-term outcome after PICU admission. Nevertheless, the increasing wealth of clinical data produced by medical devices involves very long time series representing a great number of characteristics with potential complex inter-relations that are relevant for outcome. Therefore, novel data sources challenge conventional statistical methods, such as linear regression, which are not suitable to handle larger numbers of predictors and have limited potential to capture complex relations between predictors and outcome. Compared to conventional statistics, machine learning has great potential to capture this complexity thanks to the capability to process vast amounts of data and model non-linear and highly complex interactions [21]. Machine learning is a rapidly growing field of artificial intelligence that is increasingly applied in health care settings [22–25]. Given the large number of factors and mechanisms that have been implicated on the long-term neurocognitive outcome of critically ill patients, machine learning may have added value compared to linear regression to improve neurocognitive outcome prediction. However, the value of machine learning in investigating the relation between PICU admission and long-term neurocognitive outcome has not been investigated thus far and is therefore currently unclear.

This study aims (1) to elucidate the potential relevance of patient and PICU-related characteristics for long-term adverse neurocognitive outcome after PICU admission for bronchiolitis, and (2) to perform a preliminary exploration of the potential of machine learning as compared to linear regression to improve neurocognitive outcome prediction in a relatively small sample of children after PICU admission.

## Materials and methods

### Participants

This cross-sectional observational study assessed children aged 6–12 years with a history of PICU admission during infancy (age ≤ 1 year) for bronchiolitis requiring invasive mechanical ventilation (“patient group”). The diagnosis of bronchiolitis was defined as clinical symptoms of a lower respiratory tract infection including a positive viral nasopharyngeal aspirate. The patient group was compared to normally developing peers who had not been admitted to the PICU during their life (“control group”) on neurocognitive functioning. All participants were required to be proficient in the Dutch language. We included children from the age of six, as by that age the full range of neurocognitive functions can be assessed. We focused on children of primary school age, in an attempt to limit the heterogeneity in the development of the children. Exclusion criteria were as follows: developmental disorders known to impact on neurocognitive development; physical conditions and/or behavioral deficits interfering with the ability to adequately perform neurocognitive testing; clinical signs of neurological complications during PICU admission (e.g., seizure, encephalitis, meningitis); presence of family conflict interfering with study participation (e.g., child abuse, child being placed under external supervision); and living abroad. We specifically focused on children with previous PICU admission for bronchiolitis, because this is a relative homogenous group with single organ failure that seldom manifests neurologically [26, 27] and is therefore not expected to affect neurocognitive functioning in itself.

The patient group was retrospectively recruited from a consecutive cohort admitted between 2007 and 2013 to the PICU of the Amsterdam University Medical Centers (UMC), The Netherlands. All children in the patient group received similar treatment per local clinical protocol at time of PICU admission, including invasive mechanical ventilation modes, primary and secondary choice of sedative drugs during mechanical ventilation, oxygen therapy, and nutrition. The control group was recruited through the patient group (friends and relatives) and through primary schools in The Netherlands. We aimed to include at least 64 children in the patient group and 64 children in the control group, in order to achieve sufficient statistical power to detect medium-sized group differences (Cohen’s *d* = 0.5, assuming power = 80% and alpha = .05).

### Measures

#### Patient and PICU-related characteristics

Data on socioeconomic status, past breastfeeding, mother’s smoking, and drinking of alcohol during pregnancy were collected using a parental questionnaire, as these characteristics have potential influence on children’s neurocognitive functioning [28–30]. Socioeconomic status was defined as the average level of education for the available parent(s) and/or caregiver(s) and measured with the Education Categorization Standard developed by the Statistics Netherlands [31]. This standard assesses parental education on an eight-point interval scale ranging from 1 (no education) to 8 (postdoctoral education). In case patients only had one parent, socioeconomic status was determined by the level of parental education from only that parent. Furthermore, we extracted the following patient and PICU-related characteristics from the medical files (paper files and/ or electronic clinical information system MetaVision *i*MD*soft*): sex, age, gestational age, birth weight, age and weight at PICU admission, Pediatric Index of Mortality 2 (PIM 2) score [32], duration of invasive mechanical ventilation, length of PICU stay, need for reintubation, cardiopulmonary resuscitation, use of antibiotics during PICU stay, readmission to the PICU, and the isolation of type(s) of viral agents from the nasopharyngeal aspirate. In case gestational age and birth weight had not been recorded in the medical file of the Amsterdam UMC, this information was requested from the hospital where the child was born. In addition, mechanical ventilation parameters provide information about the disease severity of the children and may therefore be relevant for their neurocognitive outcome, and also mechanical ventilation itself is potent to induce injury (e.g., to the lungs [33]). Therefore, we extracted the data of the mechanical ventilator that were hourly validated by the nurse: fraction of inspired oxygen (FiO_2_), positive inspiratory pressure (PIP), positive end-expiratory pressure (PEEP), mean airway pressure, oxygen saturation (SpO_2_), end-tidal carbon dioxide (etCO_2_), and SpO_2_/FiO_2_ ratio. At last, we extracted the laboratory measures of serum glucose, pH, partial pressure of carbon dioxide (pCO_2_), and lactate. Arterial and/ or capillary (in case patients did not have an arterial line) measures were extracted. In case patients were readmitted to the PICU, the PICU-related characteristics were collected from all PICU admissions together. After extraction of all characteristics (“raw data”), clinically relevant values were calculated (e.g., mean value, values below or above clinical cut-offs) [34]. Online Resource 1 displays the clinically relevant values of all extracted patient and PICU-related characteristics.

#### Long-term neurocognitive functioning

Neurocognitive functioning was assessed in our previous study [35] and was determined by assessment of full-scale intelligence quotient (FSIQ) and specific domains of neurocognitive functioning by a standardized and computerized neurocognitive test-battery. FSIQ was assessed to capture general neurocognitive functioning and was measured by a short form of the Wechsler Intelligence Scale for Children—Third edition (WISC-III) involving two subtests measuring verbal IQ and two scales measuring performance IQ, i.e., the subtests Vocabulary, Arithmetic, Block Design, and Picture Arrangement. FSIQ estimated with this short form has excellent validity (*r* = .95) and reliability (*r* = .90) in the normative population as well as in a mixed neurological population (*r* = .86 and *r* = .96, respectively) [36, 37]. The neurocognitive test-battery measures a broad range of key neurocognitive domains and contains a composition of child-friendly tests based on well-known neuroscientific paradigms with established validity and reliability, i.e., the Attention Network Test [38], Multisensory Integration Task [39], Tower of London [40], Rey Auditory Verbal Learning Test [41], Digit Span task [42], Klingberg task [43], and the Track & Trace task [44]. For more information, see Online Resource 2. The neurocognitive data derived from the test-battery were subjected to a pre-processing pipeline to construct neurocognitive domain scores [35]. This procedure resulted in ten neurocognitive domains that explained 78% of the variance contained in the original neurocognitive data derived from the test-battery, i.e., speed and attention, set shifting, verbal memory, visuomotor integration, verbal working memory, interference control, visual processing speed, visual working memory, planning time, and multisensory integration. Higher scores on each of the domains reflect better performance.

### Procedure

Participating children underwent neurocognitive testing by trained examiners in a quiet room with an approximate duration of 3 h, including breaks. Block randomized order of test administration was applied to counterbalance the systematic influence of fatigue on test performance.

### Pre-processing of patient and PICU-related characteristics

Missing values at random (< 10% missing values per variable) were imputed using multiple imputations [45]. Outliers (mean ± 3 SD) were winsorized [46, 47]. In order to avoid that the final model would be overly sensitive to variables with low prevalence, variables with fewer than 10 occurrences per event were eliminated. In the case of multicollinearity between variables (based on variance inflation factor > 10 and/or Pearson > 0.7 or < −0.7), the variable with the lowest correlation to FSIQ was eliminated.

### Statistical analysis

Statistical analysis was conducted using R [48], RStudio [49], the car package [50], and the caret package [51]. In order to gain insight in the association between predictor variables (patient and PICU-related characteristics) and long-term neurocognitive outcome, we selected two widely adopted machine learning algorithms Regression Trees and k-Nearest Neighbors that provide interpretable outcomes. We used multivariable linear regression analysis with backward elimination as a reference model (*p* out > 0.05). With each of the techniques (i.e., Regression Trees, k-Nearest Neighbors, Linear Regression), one model was fitted for each of the neurocognitive outcomes.

The goal of machine learning is to predict an outcome based on patterns present in the input data (training). In order to train a model to predict unseen (“new”) data, the original dataset was split into a training set (90% of the data) and a blind test set (10% of the data), which were identical for each model. The training set was then further divided into ten (folds) for five-repeated ten-fold cross-validation [52], which was used for performance validation. Each model was trained on data of the training set (nine out of ten folds), validating training performance on the tenth fold. Based on the results from model training, the mean performance across all folds was reported. Thereafter, the blind test set was used only once for each model, in order to assess internal model generalizability and model performance on data that were not used for model training. Internal model generalizability (i.e., stability of model performance on data that were not used to develop the model) was assessed by comparing model performance (the explained variance, *R*^2^) in the training set (average across folds) to the blind test set using 95% bootstrap confidence intervals (95%-CI). In case the mean *R*^2^ of the training set was within the 95%-CI of the *R*^2^ of the blind test set, we concluded that the model had sufficient internal generalization from the training data to the blind test data. Subsequently, model performance was based on the *R*^2^ in the blind test set. To assess the added value of the machine learning models as compared to our reference model, we compared the *R*^2^ of the blind test set between models, using the 95%-CI of the multivariable linear regression models as reference. For details regarding the machine learning algorithms, see Online Resource 3. All statistical testing was two-sided, α was set at .05.

## Results

### Participants

**Fig. 1:**
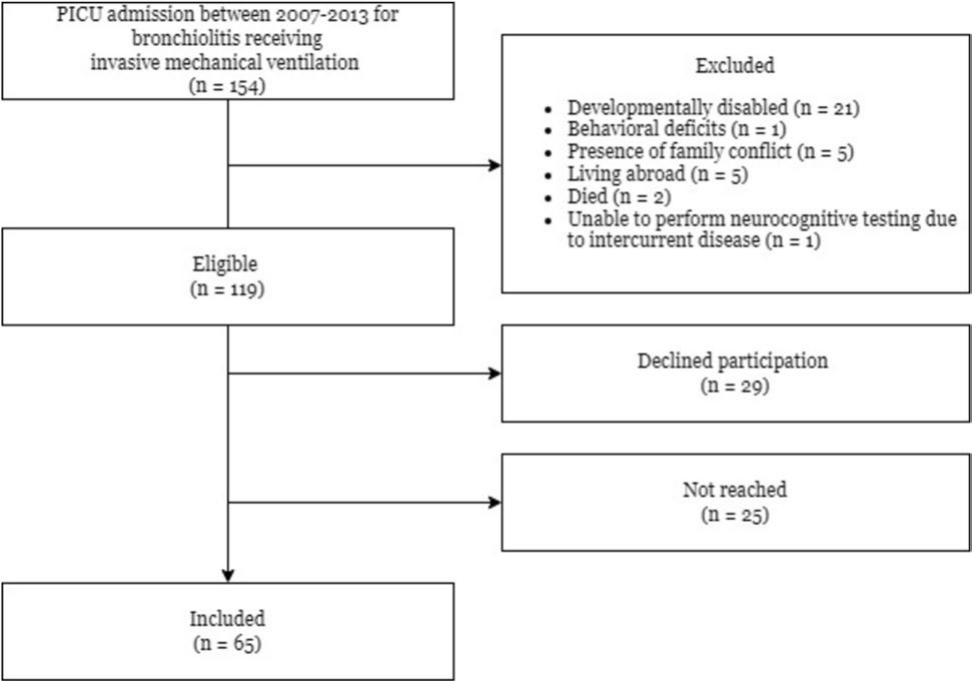
Flowchart of included children. Note: Reasons to decline participation were: not interested (*n* = 11), no time (*n* = 10), too high a burden on child (*n* = 6) or language barrier of parents (*n* = 2). Two children died due to persistent refractory pulmonary hypertension triggered by a viral infection

Children included in the patient group (*n* = 65, Fig. 1) did not differ from the total recruitment cohort of children satisfying the inclusion criteria (*n* = 119) in terms of sex, age at PICU admission, duration of mechanical ventilation, and length of PICU stay (Online Resource 4). In addition, comparison between children included in the patient group (*n* = 65) versus those eligible but not included (*n* = 54) also showed no significant differences regarding these characteristics (Online Resource 5), indicating no evidence for selection bias in the study sample. No differences between the patient and control group (*n* = 76) were found regarding sex, age, and socioeconomic status (Online Resource 6), indicating no evidence for a confounding role of demographic differences between groups. The study sample consists out of only unique patients, of which two had two PICU admissions for bronchiolitis, and five children were readmitted because of subglottic stenosis due to upper airway injury by endotracheal intubation. For these children, post-PICU time was used from the first PICU stay and PICU-related characteristics were based on all admissions together. Table 1 shows the patient and PICU-related characteristics of the included children that were used for the prediction models. Characteristics with an asterisk were eliminated in the Linear Regression and k-Nearest Neighbors models due to multicollinearity. Characteristics with less than ten occurrences per event were eliminated in all models and are only shown in Online Resource 1.

**Table 1:** Patient and PICU-related characteristics that were used for the prediction models

**Patient and PICU-related characteristics**	**Mean (SD), median (IQR), or number (%)**
Age at follow-up (years), mean (SD)	8.1 (1.2)
Sex (female), *n* (%)	26 (40.0)
Socioeconomic status, mean (SD)	5.3 (1.2)
Gestational age (weeks), median (IQR)^a^	38.1 (36.3–39.9)
Birth weight (grams), mean (SD)	3083 (968)
Breastfed in past, *n* (%)	42 (64.6)
Age at PICU admission (days), median (IQR)^a^	43.0 (23.5–79.5)
Weight at PICU admission (grams), mean (SD)	4634 (1662)
PIM 2 score, median (IQR)	1.4 (1.1–2.1)
Duration of invasive mechanical ventilation (hours), mean (SD)	169.5 (88.6)
Length of PICU stay (days), median (IQR)^a^	7.4 (5.7–9.0)
Glucose (mmol/L) during PICU admission, mean (SD)^a^	6.1 (0.8)
Episodes of glucose > 10 mmol/L, median (IQR)	0.0 (0.0–1.0)
Episodes of pCO_2_ > 6.4 kPa, median (IQR)^a^	12.0 (7.5–19.5)
Episodes of pCO_2_ < 4.7 kPa, median (IQR)	1.0 (0.0–2.0)
Episodes of pH > 7.45, median (IQR)	6.0 (4.0–11.5)
Episodes of pH < 7.35, median (IQR)	2.0 (0.0–4.0)
Episodes of SpO_2_ < 90%, median (IQR)	1.0 (0.0–2.0)
Episodes of SpO_2_ < 85%, median (IQR)^a^	0.0 (0.0–1.0)
Minimum FiO_2_ (%), median (IQR)	26.0 (25.0–30.0)
Maximum FiO_2_ (%), mean (SD)	88.6 (17.0)
Mean SpO_2_/FiO_2_ ratio, mean (SD)	2.5 (0.5)
Minimum SpO_2_/FiO_2_ ratio, mean (SD)^a^	1.1 (0.3)
Episodes of etCO_2_ < 3.5 kPa, median (IQR)	1.0 (0.0–4.0)
Episodes of etCO_2_ > 6.5 kPa, median (IQR)	5.0 (1.0–14.0)
Difference between PIP and PEEP (cmH_2_O), mean (SD)	15.9 (2.5)
Mean airway pressure (cmH_2_O), mean (SD)^a^	13.4 (1.8)

*CPR* cardiopulmonary resuscitation, *etCO*_*2*_ end-tidal carbon dioxide, *ECMO* extracorporeal membrane oxygenation, *FiO*_*2*_ fraction of inspired oxygen, *PEEP* positive end-expiratory pressure, *PICU* pediatric intensive care unit, *PIP* positive inspiratory pressure, *PIM2 score* Pediatric Index of Mortality 2 score, *PIP* positive inspiratory pressure, *SpO*_*2*_ oxygen saturation

^a^Variable eliminated in the Linear Regression and k-Nearest Neighbors models due to multicollinearity

### Neurocognitive functioning

Neurocognitive outcomes are fully described elsewhere [35] and summarized in Online Resource 7. In brief, the patient group had significantly lower FSIQ (*M* = 95.3, SD = 15.9) than the control group (*M* = 105.1, SD = 15.1; *p* < .001, Cohen’s *d* = −0.59), and significantly poorer performance on the domains Speed and Attention (*p* = .03, *d* = −0.41) and Verbal Memory (*p* < .001, *d* = −0.60). To elucidate the potential relevance of patient and PICU-related characteristics for long-term adverse neurocognitive outcome after PICU admission and to explore the potential of machine learning, these three neurocognitive outcomes were selected as outcome measures.

### Value of machine learning

#### Internal generalization

Table 2 displays the results regarding internal generalizability and performance of the models. For the majority of models, we found no significant difference in model performance on blind test data as compared to the training data, suggesting sufficient internal generalization of model performance. As exception, the Regression Trees model for Verbal Memory showed significantly higher performance in the blind test data as compared to the training data, suggesting insufficient internal generalization of model performance. The wide confidence intervals should be noted, with limited sensitivity for comparisons of internal model generalization.

**Table 2:** Cross-validated results and bootstrapped (*R* = 1000) test results

**Outcome**	**Algorithm**	***R***^**2**^ **(%) training set**	***R***^**2**^ **(%) blind test set**	**95% CI of the** ***R***^**2**^ **(%) blind test set**
FSIQ	Linear Regression	24.1	25.9	0.0, 97.3
	Regression Trees	19.7	65.3	19.2, 96.9
	k-Nearest Neighbors	8.9	15.8	0.0, 58.4
Speed and attention	Linear Regression	43.9	53.5	1.6, 98.9
	Regression Trees	31.4	70.2	12.2, 98.9
	k-Nearest Neighbors	7.6	16.9	0.0, 63.8
Verbal memory	Linear Regression	41.0	50.6	4.0, 98.5
	Regression Trees	10.8	76.7	23.0, 99.4
	k-Nearest Neighbors	7.6	16.7	0.0, 66.5

*FSIQ* full-scale intelligence quotient

#### Performance

The reference Linear Regression models showed predictive value for FSIQ (*R*^2^ = 25.9%, 95%-CI 0.0–97.3%, *p* = .005), performance on the Speed and Attention domain (*R*^2^ = 53.5%, 95%-CI 1.6–98.9%, *p* < .001) and performance on the Verbal Memory domain (*R*^2^ = 50.6%, 95%-CI 4.0–98.5%, *p* < .001). As compared to the reference Linear Regression models, we found no significant differences in performance (on blind test data) for the Regression Trees and k-Nearest Neighbors machine learning models. Again, the wide confidence intervals should be noted, reflecting limited sensitivity for model performance comparisons.

Taken together, the Regression Trees model for Verbal Memory showed poor internal generalizability of model performance to new data, while both the Regression Trees and k-Nearest Neighbors models did not reveal added value in terms of model performance as compared to Linear Regression. These findings provide no evidence for added value of these machine learning models in the prediction of long-term neurocognitive outcome.

### Prediction of long-term neurocognitive outcome

Considering that we did not find evidence for added value of the Regression Trees and k-Nearest Neighbor machine learning models, we used the Linear Regression reference models to provide insight in the variables that contribute to the prediction of long-term neurocognitive outcome (Table 3). The results show that lower FSIQ was predicted by lower birth weight and lower socioeconomic status (*R*^2^ = 25.9%, 95%-CI 0.0–97.3%). Poorer performance on the Speed and Attention domain was solely predicted by younger age at follow-up (*R*^2^ = 53.5%, 95%-CI 1.6–98.9%). Poorer performance on the Verbal Memory domain was predicted by lower birth weight, younger age at follow-up, and greater exposure to acidotic events (episodes of pH < 7.35; *R*^2^ = 50.6%, 95%-CI 4.0–98.5%).

**Table 3:** Results of the final multivariable linear regression models

**Neurocognitive outcomes**	**Predictors**	**Standardized beta**	**Unstandardized beta (95% CI)**	***p*-value**
FSIQ	*Total model*			.005
	Birth weight (grams)	0.24	0.004 (0.000, 0.008)	.047
	Socioeconomic status	0.31	4.12 (0.97, 7.28)	.011
Speed and attention	*Total model*			< .001
	Age at follow-up (years)	0.57	0.46 (0.29, 0.62)	< .001
Verbal memory	*Total model*			< .001
	Birth weight (grams)	0.44	0.001 (0.000, 0.001)	< .001
	Age at follow-up (years)	0.25	0.24 (0.04, 0.44)	.019
	Episodes of pH < 7.35	−0.29	−0.06 (−0.10, −0.02)	.008

*FSIQ* full-scale intelligence quotient

### Exploratory analysis

We further explored exposure to acidotic events (episodes of pH < 7.35). Acidosis (pH < 7.35) was observed in 47 of 65 patients (72.3%) and regarding acidosis severity, the following pH values were observed: pH 7.25–7.35, 196 observations in 47 patients; pH 7.20–7.25, 36 observations in 16 patients; pH < 7.20, 41 observations in 10 patients. In 247 (90.5%) observations, acidosis co-occurred with elevated pCO_2_, in one observation with elevated lactate, and in five observations with a combination of elevated pCO_2_ and elevated lactate. In 235 (86%) observations of acidosis, lactate was not measured. The pattern findings suggest a respiratory origin is more likely to explain the occurrence of acidosis as compared to a metabolic origin, although a combination cannot be ruled out due to the unavailability of lactate measurements for the majority of acidotic events.

The relation between verbal memory outcome and other aspects of acidosis exposure was also explored by multivariable linear regression analysis with backward elimination. The following independent pH variables were used: lowest pH value of each patient, mean pH value of each patient, and exposure to severe acidotic events (pH < 7.20). In addition, we also used exposure to hypercapnia (pCO_2_ > 6.4 kPa) as an independent variable. Results are displayed in Table 4. Lower mean pH values and greater exposure to elevated pCO_2_ levels were associated with poorer verbal memory outcome (*p* = .038 and *p* = .011, respectively).

**Table 4:** Exploratory analysis regarding acidotic events

**Neurocognitive outcome**	**Predictors**	**Median (IQR)**	**Standardized beta**	**Unstandardized beta (95% CI)**	***p*-value**
Verbal memory	Lowest pH value for each patient	7.29 (7.21–7.36)	0.23	2.34 (−0.16, 4.85)	.07
	Mean pH value for each patient	7.42 (7.41–7.44)	0.26	9.15 (0.52, 17.79)	.038
	Episodes of pH < 7.20	0 (0–0)	−0.17	−0.06 (−0.15, 0.03)	.21
	Episodes of pCO_2_ > 6.4 kPa	12.0 (7.5–19.5)	−0.32	−0.03 (−0.05, -0.01)	.011

*pCO*_*2*_ partial pressure of carbon dioxide

## Discussion

This study aimed (1) to elucidate the potential relevance of patient and PICU-related characteristics for long-term adverse neurocognitive outcome after PICU admission for bronchiolitis, and (2) to perform a preliminary exploration of the potential of machine learning as compared to linear regression to improve neurocognitive outcome prediction in a relatively small sample of children after PICU admission. The results provide no evidence for the added value of machine learning models as compared to conventional linear regression analysis in the prediction of long-term neurocognitive outcome after PICU admission for bronchiolitis. As may be expected, linear regression analysis revealed that neurocognitive outcome was associated with demographic and perinatal characteristics (socioeconomic status, age at follow-up, and birth weight). Moreover, children with greater exposure to acidotic events during PICU admission for bronchiolitis had poorer verbal memory outcome. As the involvement of the central nervous system in the pathology of bronchiolitis is unlikely [26, 27], the relation between acidotic events and neurocognitive outcome may reflect either potentially harmful effects of acidosis itself, or reflect related processes such as hypercapnia, hypoxic, and/or ischemic events during PICU admission.

Given the large number of factors and mechanisms that have been proposed to contribute to long-term neurocognitive outcome of critically ill patients, characteristics of machine learning models (such as flexibility, ability to model non-linear relationships, more advanced inherent selection strategies) may provide potential to improve neurocognitive outcome prediction. We used machine learning in the current sample to perform a preliminary exploration of the potential value of machine learning to improve outcome prediction in a relatively smaller sample, although comparable in size to other post-PICU follow-up studies [53]. Regarding comparison of prediction models, we found no evidence for added value of the Regression Trees and k-Nearest Neighbors machine learning models as compared to conventional linear regression analysis. The wide confidence intervals, potentially reflecting the small sample size of the blind test set, provided limited sensitivity for model comparisons. Nevertheless, the findings suggest that machine learning models may not have added value in smaller sample sizes. Although there are examples of successful machine learning applications in small datasets [54], machine learning flourishes by large datasets not easily obtained in clinical settings [55]. This further stresses the importance of multicenter (international) collaborations [56] to pool clinical data and acquire larger datasets for clinical research into advanced outcome prediction using machine learning. In this study, model performance (assessed by *R*^2^) was not sufficient to have utility for individual outcome prediction. Nevertheless, it should be stressed that additional measures of model performance (e.g., precision and calibration) are critical to evaluate when evaluating the value of prediction models for individual outcome prediction [57]. In this study, we found no evidence for a typical pattern of overfitting (i.e., relatively high performance on training data combined with relatively low performance on blind test data). Conversely, a pattern of relatively high performance on the blind test data combined with relatively low performance on training data can be observed, indicating instable model performance. Considering the relatively larger number of predictors relative to the size of the study sample, more comprehensive data reduction and predictor selection methodology could decrease the amount of predictors for each model and potentially improve the performance of machine learning in future work, and is considered particularly important for smaller samples.

The results of our study further show that lower socioeconomic status was associated with lower intelligence after PICU admission for bronchiolitis. Abundant research has documented the relation between lower socioeconomic status and poorer neurocognitive functioning, of which the origin is matter of debate [18, 19, 28]. For example, poverty in early childhood and adverse environmental influences have been found related to neurocognitive functioning later in life [28]. In addition, literature shows that enriched environments throughout development influence brain plasticity and gene expression and resultant phenotypic cognitive traits [28]. We also observed that younger age at follow-up was associated with poorer neurocognitive functioning (i.e., poorer speed and attention and verbal memory). Most likely, this finding reflects a developmental effect, i.e., reflecting the commonly observed age-related improvements in neurocognitive functioning [42]. Children with younger age at follow-up also had shorter recovery time (*r* = .98), which could theoretically also have contributed to relatively poorer neurocognitive performance in younger children. Indeed, literature shows an association between younger age at follow-up and poorer neurocognitive functioning in some PICU subgroups, such as children admitted after heart- or heart-lung-transplantation [7], although contradicting findings have been reported in children and adolescents who survived meningococcal septic shock [20]. Furthermore, lower birth weight was associated with lower intelligence and poorer verbal memory. This result is consistent with existing work reporting an association between lower birth weight and poorer neurocognitive functioning [58–60].

The findings of our study further suggest that greater exposure to acidotic events during PICU admission is associated with poorer verbal memory outcome. In experimental studies, several mechanisms have been proposed that may explain a potential negative effect of acidosis on the central nervous system, such as acidosis causing denaturation of proteins and nucleic acids, triggering cell swelling potentially leading to cellular edema and osmolysis, and inhibition of excitatory neurotransmission in the hippocampus, and influencing neuronal vulnerability indirectly by damaging glial cells [61, 62]. Although the translation of these findings from the literature to our study findings is unclear, our findings indicate that acidotic events may be implicated in negative effects on the central nervous system, whether or not through other neurotoxic processes such as hypercapnia, hypoxia, or ischemia. In our exploratory analyses, we found additional evidence indicating that higher pCO_2_ measurements, compatible with a respiratory origin of acidosis, were also related to poorer verbal memory outcome. Regardless of the exact mechanisms at play, our findings suggest that children with greater exposure to acidotic events are at risk of adverse long-term neurocognitive outcome after PICU admission for bronchiolitis, a finding that awaits replication in future prospective studies.

Bronchiolitis is a relatively mild indication for PICU admission that seldom manifests neurologically [26, 27] and is therefore not expected to affect neurocognitive functioning in itself. The observed adverse long-term neurocognitive outcomes may suggest that (a combination of) secondary consequences of bronchiolitis and/or PICU treatment may negatively affect outcomes after PICU admission. In previous work, we found no evidence for a relationship between exposure to sedatives, analgesics, anesthetics (per local protocol that was used at that time at our PICU) and a range of neurocognitive outcomes in the current sample [35]. In addition, duration of invasive mechanical ventilation was also not associated with neurocognitive outcomes [35]. In recent years, PICU therapy for bronchiolitis shifted to less invasive mechanical ventilation and more high-flow nasal cannula, with potential relevance for long-term outcome. Nevertheless, we found no association between invasive mechanical ventilation and neurocognitive outcomes, suggesting that the shift towards less invasive ventilation is unlikely to influence neurocognitive outcome. Furthermore, other factors such as hypoxic episodes, hypotension associated with mechanical ventilation, and metabolic derangements may have negatively affected children’s neurocognitive outcome after PICU admission [12–14, 63]. As understanding of the exact nature and origin of difficulties in neurocognitive functioning is a prerequisite for successful prevention and intervention, the findings of our study highlight the importance of large prospective studies aimed at identifying the combination of factors that may account for adverse neurocognitive outcome in children admitted to the PICU for bronchiolitis, and for PICU admission in general.

Although prevention strategies, such as respiratory syncytial virus vaccine in pregnancy [64], show promising results, children will continue to be admitted to the PICU for bronchiolitis and for other admission indications. The findings of this study suggest that these children may be at risk of adverse neurocognitive outcome, even in the absence of a clear neurological manifestation of the underlying disease. Neurocognitive impairments are known to interfere with development in crucial outcome domains [8–11]. In addition, the results of our previous study [65], in which we investigated the same children included in the current study, showed that these children are at risk of long-term adverse daily life outcomes in terms of academic performance and health-related quality of life regarding school functioning 6–12 years after PICU admission for bronchiolitis. Furthermore, the findings of that study [65] suggest that lower intelligence may contribute to academic difficulties after PICU admission. Our findings underline the importance of long-term structured follow-up after PICU admission, even in the absence of underlying disease with neurological manifestation, enabling early identification and appropriate management of adverse outcomes. Furthermore, as it is unclear whether adverse neurocognitive outcomes can be catched up later in life, it may be warranted to continue follow-up monitoring into adulthood.

A limitation of our study is that a substantial number of eligible children (45.4%) did not participate in our study, mainly because they were not reached despite our efforts. However, we deem it unlikely that this has caused important selection bias because the study sample did not differ from the total cohort of eligible children in terms of demographic characteristics and illness severity. A second limitation relates to the operationalization of socioeconomic status as the average level of parental education. The use of parental education is only one attribute of the multifaceted construct of socioeconomic status, not accounting for the roles of, for example, income and level of professional functioning [66]. This may limit the generalizability of the study to communities with wide disparities according to for example race, ethnicity, economic opportunities, and/or insurance status. Furthermore, we acknowledge that the reported associations between risk factors and outcome may not reflect causal relationships [67]. Important to note, is that the number of acidotic events was determined on blood gas analyses measured based on clinical signs of respiratory distress. Therefore, the number of assessed blood gas analyses varied between patients based on the presentation of clinical state. In addition, we included both arterial and capillary blood gas and lactate measures, as only a minority of the children had an arterial line. Yet, capillary blood gases accurately reflect arterial pH and pCO_2_ in most PICU patients (in particular in hemodynamic stable patients) [68]. Another limitation of this study is that we did not perform external validation of the models, such as by an independently collected dataset sample of another hospital. Therefore, the hypothesis that acidotic events may increase the risk of adverse verbal memory outcome awaits replication in future work. At last, this study has modest sample size and hence had limited statistical power [69]. A strength of our study is that we extensively investigated patient and PICU-related characteristics in the relation between PICU admission and neurocognitive outcome. In addition, we focused on children admitted to the PICU for bronchiolitis, in an attempt to control for the confounding effect of underlying disease on outcome.

## Conclusion

The findings of this study suggest that in children with previous PICU admission for bronchiolitis, (1) lower birth weight, younger age at follow-up, and lower socioeconomic are associated with poorer neurocognitive outcome; and (2) greater exposure to acidotic events during PICU admission is associated with poorer verbal memory outcome. Our study does not provide evidence for the added value of machine learning models as compared to conventional linear regression analysis in the prediction of long-term neurocognitive outcome in a relatively small sample of children with PICU admission. This study further highlights the importance of structured follow-up to monitor long-term outcome of children after PICU admission.

### Supplementary Information

Below is the link to the electronic supplementary material.

Supplementary file1 (DOCX 24 KB)

Supplementary file2 (DOCX 39 KB)

Supplementary file3 (DOCX 26 KB)

Supplementary file4 (DOCX 14 KB)

Supplementary file5 (DOCX 14 KB)

Supplementary file6 (DOCX 14 KB)

Supplementary file7 (DOCX 20 KB)

## Data Availability

Further group-level data are available on request from the corresponding author. Research data at individual level are not shared due to ethical restrictions under the Dutch law.

## Abbreviations

CPR: Cardiopulmonary resuscitation
ECMO: Extracorporeal membrane oxygenation
etCO_2_: End-tidal carbon dioxide
FiO_2_: Fraction of inspired oxygen
FSIQ: Full-scale intelligence quotient
PEEP: Positive end-expiratory pressure
PICU: Pediatric intensive care unit
PIM2 score: Pediatric Index of Mortality 2 score
PIP: Positive inspiratory pressure
SpO_2_: Oxygen saturation
