# Supplementary material for: Predicting long-term neurocognitive outcome after pediatric intensive care unit admission for bronchiolitis—preliminary exploration of the potential of machine learning
Source: Eur J Pediatr. 2023 Nov 6;183(1):471–82. doi: 10.1007/s00431-023-05307-3 (PMC10857960; doi:10.1007/s00431-023-05307-3)
Supplement: Supplementary file 1 — Supplementary file1 (DOCX 24 KB) [file 431_2023_5307_MOESM1_ESM.docx]

**Predicting Long-term Neurocognitive Outcome**

**after Pediatric Intensive Care Unit Admission for Bronchiolitis -**

**Preliminary Exploration of the Potential of Machine Learning**

**European Journal of Pediatrics**

*Eleonore S.V. de Sonnaville, MD, PhD^1,2,3^; Jacob Vermeule, MSc^4^;*

*Kjeld Oostra, MSc^4^; Hennie Knoester, MD, PhD^1,3^; Job B.M. van Woensel, MD, PhD^1,3^;*

*Somaya Ben Allouch, PhD^4^;* *Jaap Oosterlaan, PhD^2,3^; Marsh Kӧnigs, PhD^2,3^*

**Affiliations:**

^1^Amsterdam UMC location University of Amsterdam, Emma Children’s Hospital, Department of Pediatric Intensive Care, Meibergdreef 9, Amsterdam, The Netherlands

^2^Amsterdam UMC location University of Amsterdam, Emma Children’s Hospital, Emma Children’s Hospital Amsterdam UMC Follow Me program & Emma Neuroscience Group, Meibergdreef 9, Amsterdam, The Netherlands

^3^Amsterdam Reproduction and Development research institute, Amsterdam, The Netherlands

^4^University of Amsterdam, Informatics Institute, Science Park 904, Amsterdam, The Netherlands

**Address correspondence to:**

Eleonore S.V. de Sonnaville, Amsterdam UMC location University of Amsterdam, Emma Children’s Hospital, Department of Pediatric Intensive Care, Follow Me program & Emma Neuroscience Group, Meibergdreef 9, 1105 AZ Amsterdam, The Netherlands. Room number H8-260. Email: e.s.desonnaville@amsterdamumc.nl, telephone: +31616264285.

| **eTable 1.** Complete list of the extracted patient and PICU-related characteristics of the included children. | | |
| --- | --- | --- |
| **Patient and PICU-related characteristics** | **Mean (SD) or median [IQR]** | **Number (%)** |
| Age at follow-up (years), mean (SD) | 8.1 (1.2) |  |
| Sex (female), n (%) |  | 26 (40.0) |
| Socioeconomic status, mean (SD) | 5.3 (1.2) |  |
| Gestational age (weeks), median [IQR] * | 38.1 [36.3-39.9] |  |
| Birth weight (grams), mean (SD) | 3083 (968) |  |
| Bronchopulmonary dysplasia, n (%) ** |  | 3 (4.6) |
| Mother cigarette smoking during pregnancy, n (%) ** |  | 6 (9.2) |
| Mother drinking of alcohol during pregnancy, n (%) ** |  | 2 (3.1) |
| Breastfed in past, n (%) |  | 42 (64.6) |
| Age at PICU admission (days), median [IQR] * | 43.0 [23.5-79.5] |  |
| Weight at PICU admission (grams), mean (SD) | 4634 (1662) |  |
| PIM 2 score, median [IQR] | 1.4 [1.1-2.1] |  |
| Duration of invasive mechanical ventilation (hours), mean (SD) | 169.5 (88.6) |  |
| Length of PICU stay (days), median [IQR] * | 7.4 [5.7-9.0] |  |
| Respiratory syncytial virus positive, n (%) ** |  | 56 (86.2) |
| Reintubation, n (%) ** |  | 4 (6.2) |
| Tracheostomy, n (%) ** |  | 2 (3.1) |
| ECMO, n (%) ** |  | 1 (1.5) |
| CPR, n (%) ** |  | 2 (3.1) |
| Readmission at the PICU, n (%) ** |  | 7 (10.8) |
| Nitric oxide, n (%) ** |  | 3 (4.6) |
| Cardiostimulants, n (%) ** |  | 5 (7.7) |
| Antibiotics, n (%) ** |  | 56 (86.2) |
| Sepsis, n (%) ** |  | 1 (1.5) |
| Septic shock, n (%) ** |  | 0 (0.0) |
| Meningitis, n (%) ** |  | 0 (0.0) |
| Glucose (mmol/L) during PICU admission, mean (SD) * | 6.1 (0.8) |  |
| Episodes of glucose < 3 mmol/L, median [IQR] ** | 0.0 [0.0-0.0] |  |
| Episodes of glucose > 10 mmol/L, median [IQR] | 0.0 [0.0-1.0] |  |
| Episodes of pCO_2_ > 6.4 kPa, median [IQR] * | 12.0 [7.5-19.5] |  |
| Episodes of pCO_2_ < 4.7 kPa, median [IQR] | 1.0 [0.0-2.0] |  |
| Episodes of pH > 7.45, median [IQR] | 6.0 [4.0-11.5] |  |
| Episodes of pH < 7.35, median [IQR] | 2.0 [0.0-4.0] |  |
| Episodes of lactate > 2.1 mmol/L, median [IQR] ** | 0.0 [0.0-1.0] |  |
| Episodes of SpO_2_ < 90%, median [IQR] | 1.0 [0.0-2.0] |  |
| Episodes of SpO_2_ < 85%, median [IQR] * | 0.0 [0.0-1.0] |  |
| Minimum FiO_2_ (%), median [IQR] | 26.0 [25.0-30.0] |  |
| Maximum FiO_2_ (%), mean (SD) | 88.6 (17.0) |  |
| Mean SpO_2_/FiO_2_ ratio, mean (SD) | 2.5 (0.5) |  |
| Minimum SpO_2_/FiO_2_ ratio, mean (SD) * | 1.1 (0.3) |  |
| Episodes of etCO_2_ < 3.5 kPa, median [IQR] | 1.0 [0.0-4.0] |  |
| Episodes of etCO_2_ > 6.5 kPa, median [IQR] | 5.0 [1.0-14.0] |  |
| Difference between PIP and PEEP (cmH_2_O), mean (SD) | 15.9 (2.5) |  |
| Mean airway pressure (cmH_2_O), mean (SD) * | 13.4 (1.8) |  |
| Note. CPR = cardiopulmonary resuscitation; etCO_2_ = end-tidal carbon dioxide; ECMO = extracorporeal membrane oxygenation; FiO_2_ = fraction of inspired oxygen; PEEP = positive end-expiratory pressure; PICU = pediatric intensive care unit; PIP = positive inspiratory pressure; PIM2 score = Pediatric Index of Mortality 2 score; PIP = positive inspiratory pressure; SpO_2_ = oxygen saturation.  There were no missing data except for the variables: mother cigarette smoking during pregnancy, mother drinking of alcohol during pregnancy, birth weight and breastfed in past (≤ 3.1% missing at random). * Variable eliminated in the Linear Regression and k-Nearest Neighbors models due to multicollinearity. ** Variable eliminated in all models due to less than 10 occurrences per event. Missing values at random were imputed using multiple imputation for the following variables (≤3.1% missing at random): mother cigarette smoking during pregnancy, mother drinking of alcohol during pregnancy, birth weight and breastfed in past. | | |
