# Supplementary material for: Predicting long-term neurocognitive outcome after pediatric intensive care unit admission for bronchiolitis—preliminary exploration of the potential of machine learning
Source: Eur J Pediatr. 2023 Nov 6;183(1):471–82. doi: 10.1007/s00431-023-05307-3 (PMC10857960; doi:10.1007/s00431-023-05307-3)
Supplement: Supplementary file 2 — Supplementary file2 (DOCX 39 KB) [file 431_2023_5307_MOESM2_ESM.docx]

**Predicting Long-term Neurocognitive Outcome**

**after Pediatric Intensive Care Unit Admission for Bronchiolitis -**

**Preliminary Exploration of the Potential of Machine Learning**

**European Journal of Pediatrics**

*Eleonore S.V. de Sonnaville, MD, PhD^1,2,3^; Jacob Vermeule, MSc^4^;*

*Kjeld Oostra, MSc^4^; Hennie Knoester, MD, PhD^1,3^; Job B.M. van Woensel, MD, PhD^1,3^;*

*Somaya Ben Allouch, PhD^4^;* *Jaap Oosterlaan, PhD^2,3^; Marsh Kӧnigs, PhD^2,3^*

**Affiliations:**

^1^Amsterdam UMC location University of Amsterdam, Emma Children’s Hospital, Department of Pediatric Intensive Care, Meibergdreef 9, Amsterdam, The Netherlands

^2^Amsterdam UMC location University of Amsterdam, Emma Children’s Hospital, Emma Children’s Hospital Amsterdam UMC Follow Me program & Emma Neuroscience Group, Meibergdreef 9, Amsterdam, The Netherlands

^3^Amsterdam Reproduction and Development research institute, Amsterdam, The Netherlands

^4^University of Amsterdam, Informatics Institute, Science Park 904, Amsterdam, The Netherlands

**Address correspondence to:**

Eleonore S.V. de Sonnaville, Amsterdam UMC location University of Amsterdam, Emma Children’s Hospital, Department of Pediatric Intensive Care, Follow Me program & Emma Neuroscience Group, Meibergdreef 9, 1105 AZ Amsterdam, The Netherlands. Room number H8-260. Email: e.s.desonnaville@amsterdamumc.nl, telephone: +31616264285.

| **eTable 2. References to the validity and reliability data of the neurocognitive test-battery** | | |
| --- | --- | --- |
| **Neurocognitive tests** | **Validity** | **Reliability** |
| Attention Network Test | [1] | [2] |
| Multisensory Integration Task | [3] | [3] |
| Tower of London | [4] | [5] |
| Rey Auditory Verbal Learning Test | [6] | [6] |
| Digit Span task | [7] | [7] |
| Klingberg task | [8] | [8] |
| Track & Trace task | [9] | [10] |

**References**

1. de Souza Almeida R, Faria-Jr A, Klein RM. On the origins and evolution of the Attention Network Tests. Neurosci Biobehav Rev. 2021;126:560-72.

2. Ishigami Y, Klein RM. Repeated measurement of the components of attention using two versions of the Attention Network Test (ANT): stability, isolability, robustness, and reliability. J Neurosci Methods. 2010;190(1):117-28.

3. Königs M, Weeda WD, van Heurn LW, Vermeulen RJ, Goslings JC, Luitse JS, Poll-The BT, Beelen A, van der Wees M, Kemps RJ, Catsman-Berrevoets CE, Oosterlaan J. Pediatric traumatic brain injury affects multisensory integration. Neuropsychology. 2017;31(2):137-48.

4. Shallice T. Specific impairments of planning. Philos Trans R Soc Lond B Biol Sci. 1982;298(1089):199-209.

5. Unterrainer JM, Rahm B, Loosli SV, Rauh R, Schumacher LV, Biscaldi M, Kaller CP. Psychometric analyses of the Tower of London planning task reveal high reliability and feasibility in typically developing children and child patients with ASD and ADHD. Child Neuropsychol. 2020;26(2):257-73.

6. Kingma A, van den Burg W. Three parallel versions of the Rey Auditory Verbal Learning Test for children Dutch version: instructions & normative data [Drie parallelversies van de 15-woordentest voor kinderen: handleiding & normering]. Stichting Kinderneuropsychologie Noord Nederland 2005.

7. Wechsler D. Wechsler Intelligence Scale for Children (3rd ed.) (WISC-III): Manual. San Antonio, TX: Psychological Corporation; 1991.

8. Nutley SB, Söderqvist S, Bryde S, Humphreys K, Klingberg T. Measuring working memory capacity with greater precision in the lower capacity ranges. Dev Neuropsychol. 2010;35(1):81-95.

9. De Kieviet JF, Stoof CJ, Geldof CJ, Smits N, Piek JP, Lafeber HN, Van Elburg RM, Oosterlaan J. The crucial role of the predictability of motor response in visuomotor deficits in very preterm children at school age. Dev Med Child Neurol. 2013;55(7):624-30.

10. Psotta R, Dostál D, Sarvestan J, Prycl D, Kašpar O, Křížová I. Evaluation of Predictive Motor Control With Two Touchscreen Tablet-Based Tests: Reliability and Validity in School-Aged Children. Percept Mot Skills. 2023;130(1):283-300.
