# Supplementary material for: Predicting long-term neurocognitive outcome after pediatric intensive care unit admission for bronchiolitis—preliminary exploration of the potential of machine learning
Source: Eur J Pediatr. 2023 Nov 6;183(1):471–82. doi: 10.1007/s00431-023-05307-3 (PMC10857960; doi:10.1007/s00431-023-05307-3)
Supplement: Supplementary file 3 — Supplementary file3 (DOCX 26 KB) [file 431_2023_5307_MOESM3_ESM.docx]

**Predicting Long-term Neurocognitive Outcome**

**after Pediatric Intensive Care Unit Admission for Bronchiolitis -**

**Preliminary Exploration of the Potential of Machine Learning**

**European Journal of Pediatrics**

*Eleonore S.V. de Sonnaville, MD, PhD^1,2,3^; Jacob Vermeule, MSc^4^;*

*Kjeld Oostra, MSc^4^; Hennie Knoester, MD, PhD^1,3^; Job B.M. van Woensel, MD, PhD^1,3^;*

*Somaya Ben Allouch, PhD^4^;* *Jaap Oosterlaan, PhD^2,3^; Marsh Kӧnigs, PhD^2,3^*

**Affiliations:**

^1^Amsterdam UMC location University of Amsterdam, Emma Children’s Hospital, Department of Pediatric Intensive Care, Meibergdreef 9, Amsterdam, The Netherlands

^2^Amsterdam UMC location University of Amsterdam, Emma Children’s Hospital, Emma Children’s Hospital Amsterdam UMC Follow Me program & Emma Neuroscience Group, Meibergdreef 9, Amsterdam, The Netherlands

^3^Amsterdam Reproduction and Development research institute, Amsterdam, The Netherlands

^4^University of Amsterdam, Informatics Institute, Science Park 904, Amsterdam, The Netherlands

**Address correspondence to:**

Eleonore S.V. de Sonnaville, Amsterdam UMC location University of Amsterdam, Emma Children’s Hospital, Department of Pediatric Intensive Care, Follow Me program & Emma Neuroscience Group, Meibergdreef 9, 1105 AZ Amsterdam, The Netherlands. Room number H8-260. Email: e.s.desonnaville@amsterdamumc.nl, telephone: +31616264285.

**Regression Trees**

Regression Trees is a non-parametric machine learning algorithm with high interpretability of outcomes.[1] In order to increase its predictive power and to reduce the risk of overfitting, the bootstrap aggregating (bagging) method was used.[2] The bagging method consists of three steps: (1) generate n bootstrapped samples out of the training dataset; (2) train a regression tree from every sample; (3) take the average predictions from all trained trees. Bagging automatically leaves out 33% of the data within each sample, the out-of-bag sample, which is used for cross-validating the accuracy of the models. A hyperparameter for the bagging method is the optimal number of bagging samples to be generated, which is found by creating a hyper-grid to loop over several different combinations of hyperparameters. It generates 90 models with 10 to 100 bagging samples. Thereafter the error per model is plotted against the number of samples (as increasing the number of samples reduces the error) in order to find the n where the error stabilizes. This n is used as the number of bagging samples. Finally, the models are cross-validated and evaluated based on the performance metrics and the performance of the bagged regression trees is compared to the outcomes of the single tree. The importance of each predictor variable is rated from 0-100% and indicates for how many of the bagging samples this specific variable was used. Variable importance of the bagged Regression Trees in this study is displayed in eTable 3.

| **eTable 3.** Variable importance of the bagged Regression Trees | | |
| --- | --- | --- |
| **Neurocognitive outcomes** | **Predictor variables** | **Variable importance (%)** |
| **FSIQ** |  |  |
|  | Birth weight (grams) | 100 |
|  | Mean difference between PIP and PEEP (cmH_2_O) | 89.6 |
|  | Socioeconomic status | 79.3 |
|  | Minimum SpO_2_/FiO_2_ ratio | 69.4 |
|  | Mean airway pressure (cmH_2_O) | 65.6 |
|  | Episodes of pH > 7.45 | 65.3 |
|  | Mean SpO_2_/FiO_2_ ratio | 64.4 |
|  | Glucose (mmol/L) | 64.2 |
|  | Duration of invasive mechanical ventilation (hours) | 63.0 |
|  | Gestational age (weeks) | 58.7 |
|  | Age at follow-up (years) | 47.9 |
|  | Length of PICU stay (days) | 44.8 |
|  | Weight at PICU admission (grams) | 42.7 |
|  | Episodes of pCO_2_ > 6.4 kPa | 34.0 |
|  | Episodes of etCO_2_ > 6.5 kPa | 33.1 |
|  | PIM 2 score | 30.8 |
|  | Age at PICU admission (days) | 26.3 |
|  | Episodes of etCO_2_ < 3.5 kPa | 15.3 |
|  | Episodes of pH < 7.35 | 13.3 |
|  | Maximum FiO_2_ (%) | 12.7 |
|  | Sex | 12.1 |
|  | Episodes of SpO_2_ < 85% | 10.1 |
|  | Episodes of SpO_2_ < 90% | 9.2 |
|  | Episodes of glucose > 10 mmol/L | 5.9 |
|  | Breastfed in past | 0.7 |
|  | Episodes of pCO_2_ < 4.7 kPa | 0.5 |
|  | Minimum FiO_2_ (%) | 0.0 |
| **Speed and attention** |  |  |
|  | Age at follow-up (years) | 100 |
|  | Mean airway pressure (cmH_2_O) | 48.7 |
|  | Duration of invasive mechanical ventilation (hours) | 47.0 |
|  | Birth weight (grams) | 45.1 |
|  | Mean difference between PIP and PEEP (cmH_2_O) | 45.1 |
|  | PIM 2 score | 42.4 |
|  | Glucose (mmol/L) | 41.4 |
|  | Episodes of etCO_2_ > 6.5 kPa | 39.4 |
|  | Weight at PICU admission (grams) | 37.7 |
|  | Mean SpO_2_/FiO_2_ ratio | 37.0 |
|  | Gestational age (weeks) | 31.3 |
|  | Age at PICU admission (days) | 29.9 |
|  | Minimum SpO_2_/FiO_2_ ratio | 29.3 |
|  | Episodes of pH < 7.35 | 26.6 |
|  | Socioeconomic status | 23.8 |
|  | Episodes of pCO_2_ > 6.4 kPa | 22.8 |
|  | Episodes of SpO_2_ < 90% | 16.4 |
|  | Episodes of etCO_2_ < 3.5 kPa | 15.9 |
|  | Length of PICU stay (days) | 13.1 |
|  | Maximum FiO_2_ (%) | 12.3 |
|  | Episodes of pCO_2_ < 4.7 kPa | 11.9 |
|  | Episodes of pH > 7.45 | 10.0 |
|  | Sex | 6.6 |
|  | Episodes of glucose > 10 mmol/L | 6.4 |
|  | Minimum FiO_2_ (%) | 4.3 |
|  | Episodes of SpO_2_ < 85% | 2.0 |
|  | Breastfed in past | 0.0 |
| **Verbal memory** |  |  |
|  | Birth weight (grams) | 100 |
|  | Age at follow-up (years) | 94.2 |
|  | Episodes of pCO_2_ > 6.4 kPa | 91.7 |
|  | Weight at PICU admission (grams) | 87.1 |
|  | Gestational age (weeks) | 79.4 |
|  | Age at PICU admission (days) | 73.0 |
|  | Duration of invasive mechanical ventilation (hours) | 62.2 |
|  | Episodes of pH < 7.35 | 59.1 |
|  | Episodes of etCO_2_ > 6.5 kPa | 53.5 |
|  | Glucose (mmol/L) | 53.2 |
|  | Mean airway pressure (cmH_2_O) | 53.1 |
|  | Minimum SpO_2_/FiO_2_ ratio | 50.8 |
|  | PIM 2 score | 38.7 |
|  | Mean SpO_2_/FiO_2_ ratio | 38.6 |
|  | Mean difference between PIP and PEEP (cmH_2_O) | 36.7 |
|  | Episodes of SpO_2_ < 90% | 35.9 |
|  | Socioeconomic status | 34.9 |
|  | Episodes of etCO_2_ < 3.5 kPa | 33.1 |
|  | Length of PICU stay (days) | 27.1 |
|  | Episodes of pCO_2_ < 4.7 kPa | 24.1 |
|  | Episodes of pH > 7.45 | 19.3 |
|  | Episodes of glucose > 10 mmol/L | 14.1 |
|  | Maximum FiO_2_ (%) | 13.5 |
|  | Breastfed in past | 2.7 |
|  | Sex | 0.4 |
|  | Minimum FiO_2_ (%) | 0.4 |
|  | Episodes of SpO_2_ < 85% | 0.0 |
| Note. The importance of each predictor variable is rated from 0-100% and indicates for how many of the bagging samples this specific variable was used. | | |

**k-Nearest Neighbors**

K-Nearest Neighbor is a proximity based algorithm that uses ‘feature similarity’ in order to predict the outcomes of new data that is provided to the model. The distance metric used to determine the relative closeness of the datapoint is the euclidean distance. First, the numerical variables were scaled by transformation into z-scores. Second, the categorical variables were converted to dummy coded variables (1 = presence, 0 = absence). Third, the optimal value of the number of neigbors (k) was determined by incorporating a hyperparameter tuner that searches for the optimal value for k out of 20 different values for k. After every increment of k by 1 the model was cross-validated and the performance metrics were calculated.

The outcome value for a new patient is determined by the proximity of its features to that of other patients. For example: a new patient’s value for FSIQ is determined by the weighted average of FSIQ values of the k patients whose patient and PICU-related characteristics are most similar. As k-Nearest Neighbors models are never trained, all predictors in the model are equally important to predict an outcome.

**References**

1. Breiman L, Friedman J, Stone CJ, Olshen RA. Classification and regression trees. CRC press1984.

2. Breiman L. Bagging predictors. Machine learning1996. p. 123–40.
