# Supplementary material for: Predicting long-term neurocognitive outcome after pediatric intensive care unit admission for bronchiolitis—preliminary exploration of the potential of machine learning
Source: Eur J Pediatr. 2023 Nov 6;183(1):471–82. doi: 10.1007/s00431-023-05307-3 (PMC10857960; doi:10.1007/s00431-023-05307-3)
Supplement: Supplementary file 4 — Supplementary file4 (DOCX 14 KB) [file 431_2023_5307_MOESM4_ESM.docx]

**Predicting Long-term Neurocognitive Outcome**

**after Pediatric Intensive Care Unit Admission for Bronchiolitis -**

**Preliminary Exploration of the Potential of Machine Learning**

**European Journal of Pediatrics**

*Eleonore S.V. de Sonnaville, MD, PhD^1,2,3^; Jacob Vermeule, MSc^4^;*

*Kjeld Oostra, MSc^4^; Hennie Knoester, MD, PhD^1,3^; Job B.M. van Woensel, MD, PhD^1,3^;*

*Somaya Ben Allouch, PhD^4^;* *Jaap Oosterlaan, PhD^2,3^; Marsh Kӧnigs, PhD^2,3^*

**Affiliations:**

^1^Amsterdam UMC location University of Amsterdam, Emma Children’s Hospital, Department of Pediatric Intensive Care, Meibergdreef 9, Amsterdam, The Netherlands

^2^Amsterdam UMC location University of Amsterdam, Emma Children’s Hospital, Emma Children’s Hospital Amsterdam UMC Follow Me program & Emma Neuroscience Group, Meibergdreef 9, Amsterdam, The Netherlands

^3^Amsterdam Reproduction and Development research institute, Amsterdam, The Netherlands

^4^University of Amsterdam, Informatics Institute, Science Park 904, Amsterdam, The Netherlands

**Address correspondence to:**

Eleonore S.V. de Sonnaville, Amsterdam UMC location University of Amsterdam, Emma Children’s Hospital, Department of Pediatric Intensive Care, Follow Me program & Emma Neuroscience Group, Meibergdreef 9, 1105 AZ Amsterdam, The Netherlands. Room number H8-260. Email: e.s.desonnaville@amsterdamumc.nl, telephone: +31616264285.

| **eTable 4.** Comparison of included children with the total sample of eligible children | | | |
| --- | --- | --- | --- |
| **Demographic and clinical characteristics** | **Patient group (n = 65)** | **Total sample  of eligible children (n = 119)** | **p-value** |
| Sex, % boys | 60.0 | 59.7 | .96 |
| Age at PICU admission (days), median [IQR] | 43.0 [23.5-79.5] | 45.0 [27.0-82.0] | .56 |
| Mechanical ventilation (days), mean (SD) | 6.6 (2.8) | 6.3 (2.7) | .27 |
| PICU stay (days), median [IQR] | 7.4 [5.7-9.0] | 6.88 [5.0-8.7] | .23 |
| Note. PICU = Pediatric Intensive Care Unit | | | |
