# Supplementary material for: Predicting long-term neurocognitive outcome after pediatric intensive care unit admission for bronchiolitis—preliminary exploration of the potential of machine learning
Source: Eur J Pediatr. 2023 Nov 6;183(1):471–82. doi: 10.1007/s00431-023-05307-3 (PMC10857960; doi:10.1007/s00431-023-05307-3)
Supplement: Supplementary file 7 — Supplementary file7 (DOCX 20 KB) [file 431_2023_5307_MOESM7_ESM.docx]

**Predicting Long-term Neurocognitive Outcome**

**after Pediatric Intensive Care Unit Admission for Bronchiolitis -**

**Preliminary Exploration of the Potential of Machine Learning**

**European Journal of Pediatrics**

*Eleonore S.V. de Sonnaville, MD, PhD^1,2,3^; Jacob Vermeule, MSc^4^;*

*Kjeld Oostra, MSc^4^; Hennie Knoester, MD, PhD^1,3^; Job B.M. van Woensel, MD, PhD^1,3^;*

*Somaya Ben Allouch, PhD^4^;* *Jaap Oosterlaan, PhD^2,3^; Marsh Kӧnigs, PhD^2,3^*

**Affiliations:**

^1^Amsterdam UMC location University of Amsterdam, Emma Children’s Hospital, Department of Pediatric Intensive Care, Meibergdreef 9, Amsterdam, The Netherlands

^2^Amsterdam UMC location University of Amsterdam, Emma Children’s Hospital, Emma Children’s Hospital Amsterdam UMC Follow Me program & Emma Neuroscience Group, Meibergdreef 9, Amsterdam, The Netherlands

^3^Amsterdam Reproduction and Development research institute, Amsterdam, The Netherlands

^4^University of Amsterdam, Informatics Institute, Science Park 904, Amsterdam, The Netherlands

**Address correspondence to:**

Eleonore S.V. de Sonnaville, Amsterdam UMC location University of Amsterdam, Emma Children’s Hospital, Department of Pediatric Intensive Care, Follow Me program & Emma Neuroscience Group, Meibergdreef 9, 1105 AZ Amsterdam, The Netherlands. Room number H8-260. Email: e.s.desonnaville@amsterdamumc.nl, telephone: +31616264285.

| **eTable 7.** Neurocognitive outcomes of children in the patient and control group | | | | | | |
| --- | --- | --- | --- | --- | --- | --- |
| **Neurocognitive outcomes** | **Description** | **Patient group,**  **Mean (SD)**  **(n = 65)** | **Control group,**  **Mean (SD)**  **(n = 76)** | **Mean (SE) difference** | **p-value *** | **Cohen’s *d*** |
| FSIQ | Intelligence | 95.3 (15.9) | 105.1 (15.1) | -8.46 (1.98) | **< .001** | -0.59 |
| ***Neurocognitive domains*** |  |  |  |  |  |  |
| Speed and attention | Speed and variability of information processing and attention | -0.19 (0.95) | 0.16 (1.02) | -0.41 (0.15) | **.03** | -0.41 |
| Set shifting | Speed of shifting between response types | -0.03 (1.03) | 0.02 (0.98) | -0.08 (0.16) | .75 | -0.08 |
| Verbal memory | Learning and memory for verbal information | -0.29 (1.13) | 0.24 (0.81) | -0.60 (0.14) | **< .001** | -0.60 |
| Visuomotor integration | Speed and flexibility of visuomotor integration | 0.13 (1.06) | -0.11 (0.93) | 0.25 (0.17) | .22 | 0.25 |
| Verbal working memory | Short-term memory and manipulation of verbal information | -0.15 (1.03) | 0.13 (0.96) | -0.27 (0.16) | .17 | -0.27 |
| Interference control | Speed of suppressing distracting information | 0.12 (1.01) | -0.11 (0.98) | 0.21 (0.16) | .25 | 0.21 |
| Visual processing speed | Speed of visual information processing | -0.03 (1.06) | 0.02 (0.95) | -0.06 (0.16) | .81 | -0.06 |
| Visual working memory | Short-term memory and manipulation of verbal information | -0.16 (1.01) | 0.13 (0.98) | -0.29 (0.17) | .17 | -0.29 |
| Planning time | Speed and capacity of planning ahead | 0.20 (0.99) | -0.17 (0.98) | 0.38 (0.16) | .05 | 0.38 |
| Multisensory integration | Accuracy for integration of information from different sensory modalities | 0.02 (1.08) | -0.02 (0.93) | 0.04 (0.17) | .82 | 0.04 |
| Note. * Correction for false discovery rate applied across neurocognitive outcomes. FSIQ = estimated full-scale intelligence quotient; SD = standard deviation; SE = standard error. The directionality of neurocognitive variables was adapted so that for all scores, higher values corresponded to better task performance. | | | | | | |
